# Misstatements, misperceptions, and mistakes in controlling for covariates in observational research

**DOI:** 10.7554/eLife.82268

**Published:** 2024-05-16

**Authors:** Xiaoxin Yu, Roger S Zoh, David A Fluharty, Luis M Mestre, Danny Valdez, Carmen D Tekwe, Colby J Vorland, Yasaman Jamshidi-Naeini, Sy Han Chiou, Stella T Lartey, David B Allison

**Affiliations:** 1 Department of Epidemiology and Biostatistics, Indiana University School of Public Health-Bloomington Bloomington United States; 2 Department of Applied Health Science, Indiana University School of Public Health-Bloomington Bloomington United States; 3 Department of Statistics and Data Science, Southern Methodist University Dallas United States; 4 University of Memphis, School of Public Health Memphis United Kingdom; DaVita Labs United States; Max Planck Institute for Biology Tübingen Germany

**Keywords:** independent variable, covariate measurement error, confounding, bias, causal effect, association

## Abstract

We discuss 12 misperceptions, misstatements, or mistakes concerning the use of covariates in observational or *nonrandomized* research. Additionally, we offer advice to help investigators, editors, reviewers, and readers make more informed decisions about conducting and interpreting research where the influence of covariates may be at issue. We primarily address misperceptions in the context of statistical management of the covariates through various forms of modeling, although we also emphasize design and model or variable selection. Other approaches to addressing the effects of covariates, including matching, have logical extensions from what we discuss here but are not dwelled upon heavily. The misperceptions, misstatements, or mistakes we discuss include accurate representation of covariates, effects of measurement error, overreliance on covariate categorization, underestimation of power loss when controlling for covariates, misinterpretation of significance in statistical models, and misconceptions about confounding variables, selecting on a collider, and p value interpretations in covariate-inclusive analyses. This condensed overview serves to correct common errors and improve research quality in general and in nutrition research specifically.

## Introduction

In observational or *nonrandomized* research, it is common and often wise to control for certain variables in statistical models. Such variables are often referred to as *covariates*. Covariates may be controlled through multiple means, such as inclusion on the ‘right-hand side’ or ‘predictor side’ of a statistical model, matching, propensity score analysis, and other methods (Cochran and Rubin, 1961; Streeter et al., 2017). Authors of observational research reports will frequently state that they controlled for a particular covariate and, therefore, that bias due to (often phrased as ‘confounding by’) that covariate is not present (Box 1). However, authors may write ‘We controlled for…’ when in fact they did not because of common misstatements, misperceptions, and mistakes in controlling for covariates in observational research.

Box 1. “Since both tickets had an equal probability of winning the same payoff, uncertainty about the true value of the goods exchanged could not confound results.” (Arlen and Tontrup, 2015)

“In our study population, NSAIDs other than Aspirin was not associated to PC risk and, therefore, could not confound result.” (Perron et al., 2004)

“Most of the demographic, social, and economic differences between patients in different countries were not associated significantly with acquired drug resistance and, therefore, could not confound the association.” (Cegielski et al., 2014)

“Furthermore, study time, as well as self-expectation regarding educational achievements (another potential confounder), could be controlled in the IV models. Therefore, these potential channels could not confound our analysis.” (Shih and Lin, 2017)

Herein, we describe these multiple misperceptions, misstatements, and mistakes involving the use of covariates or control variables. We have discussed misperceptions that in our collective years of experience as authors, reviewers, editors, and readers, in areas including but not limited to aging and geroscience, obesity, nutrition, statistical teaching, cancer research, behavioral science, and other life-science domains have observed to prevail in the literatures of these fields. Determining the frequency with which these misperceptions are held would require very extensive and rigorous survey research. Instead, we offer them as those we find pertinent and readers may decide for themselves which they wish to study. We now make this clear in the manuscript. Some terms we use are defined in the Glossary in Box 2. Because of the critical role of attempting to minimize or eliminate biases in association and effect estimates derived from observational research, as recently pointed out elsewhere (Brown et al., 2023), we primarily focus on misperceptions, misstatements, or mistakes leading to decisions about whether and how to control for a covariate that fails to actually control for and minimize or eliminate the possibility of bias. We also consider other errors (Brenner and Loomis, 1994) in implementation, interpretation, and understanding around analyses that involve covariate adjustment.

Box 2. **Terminology/Glossary**

**Bias.** Here, we define bias as either bias in coefficients in a model or bias in frequentist statistical significance tests. Frequentist statistical significance tests, or the ordinary tests of statistical significance using p values, are commonly reported in this journal and are described more fully here (Mayo and Cox, 2006). Under the null hypothesis that there is no true association or effect to detect in a situation, a proper unbiased frequentist test of statistical significance with continuous data and a continuous test statistic yields a uniform sampling distribution of p values (i.e. rectangular) on the interval zero. The distribution is such that the probability of observing any p value less than or equal to a, where a is the preset statistical significance level (i.e. most often 0.05), is a itself. Any statistical significance test that does not meet this standard can be said to be biased. With respect to coefficients or parameter estimates, we can say that bias is equal to the expected value of the coefficient or parameter estimate minus the actual value of the parameter or quantity to be estimated. In an unbiased estimation procedure, that quantity will be zero, meaning that the expected value of the estimate is equivalent to the value to be estimated.

**Replicability.** The National Academies of Sciences uses the following working definition for replicability: “Obtaining consistent results across studies aimed at answering the same scientific question, each of which has obtained its own data” (National Academies of Sciences, Engineering, and Medicine, 2019).

**Reproducibility.** The National Academies of Sciences uses the following working definition for reproducibility: “Obtaining consistent results using the same input data; computational steps, methods, and code; and conditions of analysis. This definition is synonymous with ‘computational reproducibility” (National Academies of Sciences, Engineering, and Medicine, 2019). Disqualifying reproducibility criteria include nonpublic data and code, inadequate record keeping, nontransparent reporting, obsolescence of the digital artifacts, flawed attempts to reproduce others’ research, and barriers in the culture of research (National Academies of Sciences, Engineering, and Medicine, 2019).

**Confounder.** There are many definitions of confounder and not all are equivalent. One definition is “(…) A pre-exposure covariate C [can] be considered a confounder for the effect of A on Y if there exists a set of covariates X such that the effect of the exposure on the outcome is unconfounded conditional on (X, C) but for no proper subset (X, C) is the effect of the exposure on the outcome unconfounded given the subset. Equivalently, a confounder is a member of a minimally sufficient adjustment set” (VanderWeele and Shpitser, 2013).

**Collider**. “A collider for a certain pair of variables is any variable that is causally influenced by both of them” (Rohrer, 2018).

**Covariate**. We utilize the word covariate to indicate a variable which could, in principle, be included in a statistical model assessing the relations between an independent variable (IV) and a dependent variable (DV).

**Residual.** The difference between the observed and fitted value of the outcome (Bewick et al., 2003).

**Independent Variable.** “Independent variables (IVs) generally refer to the presumed causes that are deliberately manipulated by experimenters” (Chen and Krauss, 2005) or observed in non-interventional research.

**Dependent Variable.** “Dependent variables (DVs) are viewed as outcomes that are affected by the independent variables” (Chen and Krauss, 2005).

**Association.** Two variables are associated when they are not independent, i.e., when the distribution of one of the variables depends on the level of the other variable (Hernán, 2004).

**Related.** We say that two variables are related; when the distribution of one variable depends on the level of the other variable. In this context, we use the words ‘related’, ‘associated’, and ‘dependent’ as interchangeable and a complement of independent (Dawid, 1979).

**[The 4 highlighted variables merit different and better definitions] Causal effect**: “A difference between the counterfactual risk of the outcome had everybody in the population of interest been exposed and the counterfactual risk of the outcome had everybody in the population been unexposed” (Hernán, 2004).

**Statistical Model**: A model used to represent the data-generating process embodying a set of assumptions, and including the uncertainties about the model itself (Cox, 2006).

**Precision**: How dispersed the measurements are between each other (ISO, 1994).

**Mediator:** Variable that is on the causal pathway from the exposure to outcome (VanderWeele and Vansteelandt, 2014).

*We have used some definitions as phrased in this glossary in some of our other manuscripts currently under review, published, or in-press articles.

We sometimes use the words *confound*, *confounder*, *confounding*, and other variants by convention or for consistency with the literature we are citing. However, because of the difficulty and inconsistency in defining confounding (Pearl and Mackenzie, 2018), we will minimize such use and try to refer primarily to potentially biasing covariates (PBCs). We define PBCs as variables other than the independent variable (IV) or dependent variable (DV) for which decisions about whether and how to condition on them, including by incorporation into a statistical model, can affect the extent to which the expected value of the estimated association of the IV with the DV deviates from the causal effect of the IV on the DV.

## Misperception 1. Construct validity

Simply because we believe an observed variable (e.g. highest educational degree earned) is a measure of a construct (e.g. socioeconomic status), it does not mean that that the observed variable accurately measures that construct or that it has sufficient validity for the elimination of it as a source of covariation biasing estimation of a parameter. This scenario is a misperception attributed to construct validity, which is defined as the extent to which a test or measure accurately measures what it is intended to measure. This misperception is conceptually defined as the assumption that a measure or set of measures accurately measures the outcome of interest; however, associations between tested variables may not adequately or appropriately represent the outcome of interest. This specific type of construct validity is perhaps best exemplified through the use of proxy variables, or variables believed to measure a construct of interest while not necessarily holding a strong linear relationship with that construct. In psychology, the Patient Health Questionaire-9 (PHQ-9) is a highly reliable, nine-item psychological inventory for screening, diagnosing, and monitoring depression as an internalizing disorder. Although these nine items have been extensively tested as an appropriate measure for depression and other internalizing disorders (Bell, 1994), it is not uncommon for researchers to modify this scale for shorter surveys (Poongothai et al., 2009). However, because the PHQ-9 has been empirically tested with a specific item set, any modification may not effectively measure depressive symptomology as accurately as when the PHQ-9 is used as intended. This problem is also salient in nutritional epidemiology for food categorization (Hanley-Cook et al., 2023). For example, ongoing debate remains about ‘food addiction’ as a measurable construct despite limited evidence to suggest such a phenomenon exists and can be empirically measured (Fletcher and Kenny, 2018).

### Why misperception 1 occurs

This misperception persists simply because issues with construct validity are difficult to identify. First, owing to continuous scientific innovations, we are finding new ways to measure complex behaviors. However, the production of new instruments or tests remains greatly outpaced by such innovation. As such, scientists may rely on old, established instruments to measure problems germane to the 21^st^ century. However, the use of these instruments has not been tested in such scenarios, i.e., measuring screentime as a predictor/construct/measure of depression and other internalizing disorders. Second, although it is easy to create a new test or instrument, testing the instrument to ensure construct validity is time-consuming and tedious. If a new instrument is not tested, then no certainty exists as to whether the construct measures what it is intended to measure. Additionally, outcomes measured from old, adapted, and new measures may only be marginally incorrect. Thus, any ability to identify unusual metrics or outcomes becomes impeded, allowing this misperception to continue.

### How to avoid misperception 1

We offer two practical recommendations to avoid this misperception. First, if using an established test or instrument that measures many constructs, then the instrument should be used in its entirety. Any alteration to the instrument (particularly relating to question wording, format, and question omission) may alter response patters to a large enough degree that the construct no longer appropriately measures what it is intended to measure. However, in cases where measures are adapted, tailored to specific populations, or created anew, the instrument will ideally be empirically tested using a variety of psychometric analyses (e.g. confirmatory factor analysis) to compare factor weights and loadings between new and adapted measures. Ideally, adaptations to an existing instrument will perform the same such that scores reflect the outcome of interest equally across versions. Other options beyond a confirmatory factor analysis include test/retest reliability—a measure of how consistently a measure obtains similar data between participants—as a secondary metric to again test the reliability and validity of an instrument relative to a measured construct.

## Misperception 2. Measurement error in a covariate only attenuates associations or effect estimates and does not create apparent effects

Measurement errors can take many forms (Fuller, 2009; Carroll et al., 2006) and are not limited to random, independent, or normally distributed errors. The errors themselves may be correlated, or the errors in measurement may be correlated, with true values of the covariate or with true values of other variables or errors in other variables. The distribution of a covariate’s measurement errors, including their variance and their associations with other variables, can greatly influence the extent to which controlling for that error-contaminated covariate will reduce, increase, or have no appreciable impact on the bias of model parameter estimation and significance testing. That is, the extent to which including a PBC will eliminate, reduce, not effect, or even potentially increase bias in estimating some elements of the model is also influenced by the measurement error distributions. Indeed, a recent review by Yland et al., 2022 delineates seven ways in which even so-called ‘non-differential’ measurement error can lead to biases away from the null hypothesis in observational epidemiologic research. We do not include all of them here but refer the reader to this cogent paper.

A frequent misleading statement in the epidemiologic literature is that ‘classical’ measurement error only attenuates effects. For example, Gibson and Zezza state, “Classical measurement errors provide comfort …since they don’t cause bias if on the left-hand side, and just attenuate if on the right-hand side, giving a conservative lower bound to any estimated causal impacts” (Gibson and Zezza, 2018). That this is untrue is knowable from theory (Fuller, 2009; Carroll et al., 2006) and has been demonstrated empirically on multiple occasions. While it is well known that the presence of measurement error in simple linear regression models leads to attenuation, the influence of measurement errors in more complex statistical models depends on the outcomes and the statistical models. Therefore, measurement error and covariates, as well as outcomes, need to be considered (Tosteson et al., 1998; Buonaccorsi et al., 2000; Yi et al., 2012; Tekwe et al., 2014; Tekwe et al., 2016; Tekwe et al., 2018; Tekwe et al., 2019).

Measurement error in the covariates is often ignored or not formally modeled. This may be the result of a general lack of awareness of the consequences on estimation and conclusions drawn regarding the covariates in regression models. This may also be the result of insufficient information regarding the measurement error variance to be included in the modeling. Yet, as a field, we should move toward analyses that account for measurement error in the covariates whenever possible (Tekwe et al., 2019).

### Why misperception 2 occurs

The influence of measurement error depends on the regression model. Therefore, it cannot be generalized that measurement error always attenuates covariate effects. In some models, the presence of measurement error does lead to attenuation, while in others, it leads to inflated effects of the covariates. A simple way to think about how measurement error can lead to bias is by exploring the nature of random measurement error itself. Let us assume that the random measurement error in our covariate exists. By *random* we mean that all the errors are independent of each other and of all other factors in the model or pertinent to the model. We know that under such circumstances, the variance in the measured values of the covariate will simply be the sum of the true variance of the construct the covariate represents plus the variance of the random measurement errors. As the ratio of the variance of the random errors over the variance of the true construct approaches infinity, the proportion of variance due to the true value of the construct approaches zero and the covariate itself is effectively nothing more than random noise. For example, we wouldn’t expect that simply controlling for the random noise generated from a random number generator would reduce the bias of the IV–DV relationship from any PBC. Although this is an extreme and exaggerated hypothetical, it makes the point that the greater the error variance, the less that controlling for the covariate actually controls for the PBC of interest. Because we know that many PBCs in the field of nutrition and obesity, perhaps most notably those involving self-reported dietary intake, are measured with error, we cannot assume that when we have controlled for a covariate, we have eliminated its biasing influence. If we allow for the possibility—indeed the virtual certainty (Dhurandhar et al., 2015; Gibney, 2022; Gibney et al., 2020)—that the errors are not all random but in some cases will be correlated with important factors in the model, then ‘all bets are off.’ We cannot predict what the effect on the model will be and the extent to which biases will be created, reduced, or both by the inclusion of such covariates without fully specifying the nature of the error structure relative to the model.

### How to avoid misperception 2

One way to reduce the concerns of such measurement error is through measurement error correction methods. Fully elucidating them is beyond the scope of this article, but thorough discussions are available (Fuller, 2009). Of course, the best way of dealing with measurement error is not to have it, but that is unachievable, particularly in observational studies. Nevertheless, we should continue to strive for ever better measurements in which measurement error is minimized (Westfall and Yarkoni, 2016) to levels that plausibly have far less biasing capacity.

## Misperception 3 (two parts)

### Misperception 3a. Continuous covariates divided into polychotomous categories for better interpretation are still well-controlled

#### Why misperception 3a occurs

Another way in which controlling for PBCs can fail involves the intersection of residual confounding and nonlinearity discussed later (see Misperception 5B).

An astute investigator may recognize the potential for nonlinearity and, therefore, choose to allow for nonlinear effects or associations of the covariate with the outcome by breaking the covariate into categories that could also allow for easier interpretation (Blas Achic et al., 2018).

This is most commonly done through the use of quantiles (on a terminological note, the adjacent bins into which subjects can be placed when the covariate is ‘chopped up’ in this manner might better be termed ‘quantile-defined categories’ and not as quantiles, quintiles, quartiles, etc). The quantiles are the cut points, not the bins formed by the cutting (Altman and Bland, 1994). Yet doing so yields, as many have explained (Veiel, 1988; Fitzsimons, 2008; Hunter and Schmidt, 1990; Irwin and McClelland, 2003; Naggara et al., 2011), ‘coarse categorization’ that effectively creates additional measurement error in the covariate. This is true even if there was no measurement error to begin with, unless the true relationship between the covariate and the outcome miraculously happens to be exactly a series of step functions with the stepping occurring exactly at the points of cutting. In contrast, if the true association is more monotonic, then this categorization loses information and increases the likely residual bias (aka ‘residual confounding’). The result is an apparent control for the covariate of interest that does not truly eliminate bias from the PBC.

#### How to avoid misperception 3a

For optimal analysis, it is advisable for researchers to avoid dichotomizing continuous covariates as much as possible, as this approach may lead to unnecessary suboptimal analysis.

### Misperception 3b. Covariates categorized in coarse rather than fine categories are more reliable in the presence of measurement error

#### Why misperception 3b occurs

A similar misperception to 3 a is that in the presence of certain forms of measurement error, coarse categorization will make the covariate data more reliable because the original data cannot support fine-grained distinctions. As described by MacCallum et al., 2002:

*In questioning colleagues about their reasons for the use of dichotomization, we have often encountered a defense regarding reliability. The argument is that the raw measure of X is viewed as not highly reliable in terms of providing precise information about individual differences but that it can at least be trusted to indicate whether an individual is high or low on the attribute of interest. Based on this view, dichotomization, typically at the median, would provide a ‘more reliable’ measure*.

It may be true that for some communication purposes, data measured with low precision merit being communicated only in broad categories and not with more precise numbers. Yet, as MacCallum et al. explains after studying dichotomization (a special case or ‘the lower limit’ of polychotomization or categorization), “...the foregoing detailed analysis shows that dichotomization will result in moderate to substantial decreases in measurement reliability under assumptions of classical test theory, regardless of how one defines a true score. As noted by Humphreys, 1978, this loss of reliable information due to categorization will tend to attenuate correlations involving dichotomized variables, contributing to the negative statistical consequences described earlier in this article. To argue that dichotomization increases reliability, one would have to define conditions that were very different from those represented in classical measurement theory” (MacCallum et al., 2002).

#### How to avoid misperception 3b

Researchers are advised to refrain from dichotomizing covariates that have low reliability because this can have a negative impact on the analysis. Claiming dichotomization will improve reliability would require defining conditions that deviate significantly from classical measurement theory (MacCallum et al., 2002), which is simply difficult to verify in real application.

## Misperception 4. Controlling for a covariate reduces the power to detect an association of the IV of interest with the DV of interest

### Why misperception 4 occurs

Investigators are often reluctant to control for covariates because they believe that doing so will reduce the power to detect the association or effective interest between the IV and the DV or outcome. Therefore, if they perceive that the covariate is one that has a bivariate unadjusted correlation of zero with the IV, they may seize upon this as an opportunity to dismiss that nonsignificant covariate from further consideration. Ironically, this is the very situation in which controlling for the covariate may be most helpful for detecting a statistically significant association between the IV and the DV. This is most clearly recognized by statistical methodologists in randomized experiments or randomized controlled trials, but is frequently misunderstood by non-statistician investigators.

If a covariate is correlated (especially if it is strongly correlated) with the outcome of interest but uncorrelated with (orthogonal to in linear models) the IV (e.g. treatment assignment in a randomized experiment), then controlling for that covariate reduces residual variance in the DV without affecting the parameter estimate for the association or effect of the IV with the DV. Unless the sample size is extremely small such that the loss of a degree of freedom by including the covariate in the analysis makes an important difference (again, it rarely will in observational studies of any size), then this increases power, often quite substantially, by reducing the residual variance and thereby lowering the denominator of the F-statistic in a regression or ANOVA context or related statistics with other testing. Omission of orthogonal covariates has been well described in the literature (Allison et al., 1997; Allison, 1995). Although omission of orthogonal covariates is ‘cleanest and clearest’ in the context of randomized experiments, conditions may prevail in observational studies in which a variable is strongly related to the DV but minimally related to the IV or exposure of interest.

Such covariates are ideal to help the investigator explore his or her hypothesis, or better yet, to formally test them with frequentist significance testing methods. Doing so will increase statistical power and precision of estimation (i.e. reduced confidence intervals on the estimated associations or effects of interest).

### How to avoid misperception 4

When conducting an analysis, it is important to base the decision to control for covariates on the scientific knowledge of the problem at hand, rather than solely on the desire for a powerful test. Researchers should keep in mind that the main purpose of adjusting for covariates is to eliminate any influence of PBCs that may distort the estimate of the desired effect. To finish, we also note that including too many variables in the model can be detrimental because one runs the risk of inducing excessive multicollinearity and overfitting.

## Misperception 5 (two parts)

### Misperception 5 a. If when controlling for *X* and *Z* simultaneously in a statistical model as predictors of an outcome *Y*, *X* is significant with *Z* in the model, but *Z* is not significant with *X* in the model, then *X* is a ‘better’ predictor than *Z*

#### Why misperception 5a occurs

Investigators may also incorrectly conclude that *X* has a true causal effect on *Y* and that *Z* does not, that *X* has a stronger causal effect on *Y* than does *Z*, or that *Z* may have a causal effect on *Y* but only through *X* as a mediating variable. None of the above conclusions necessarily follow from the stated conditions. An example of a context in which these misperceptions occur was discussed recently in a podcast in which the interlocutors considered the differential associations or effects of muscle size versus muscle strength on longevity in humans (Attia, 2022). After cogently and appropriately noting the limitations of observational research in general and in the observational study under consideration in particular, the discussants pointed out that when a statistical model was used in which both muscle size and muscle strength measurements were included at the same time, muscle size was not a significant predictor of mortality rate conditional upon muscle strength, but muscle strength was a significant predictor of mortality rate conditional upon muscle size. The discussants thus tentatively concluded that muscle strength had a causal effect on longevity and that muscle size either had no causal effect, conditional upon muscle strength, or had a lesser causal effect.

**Figure 1.**
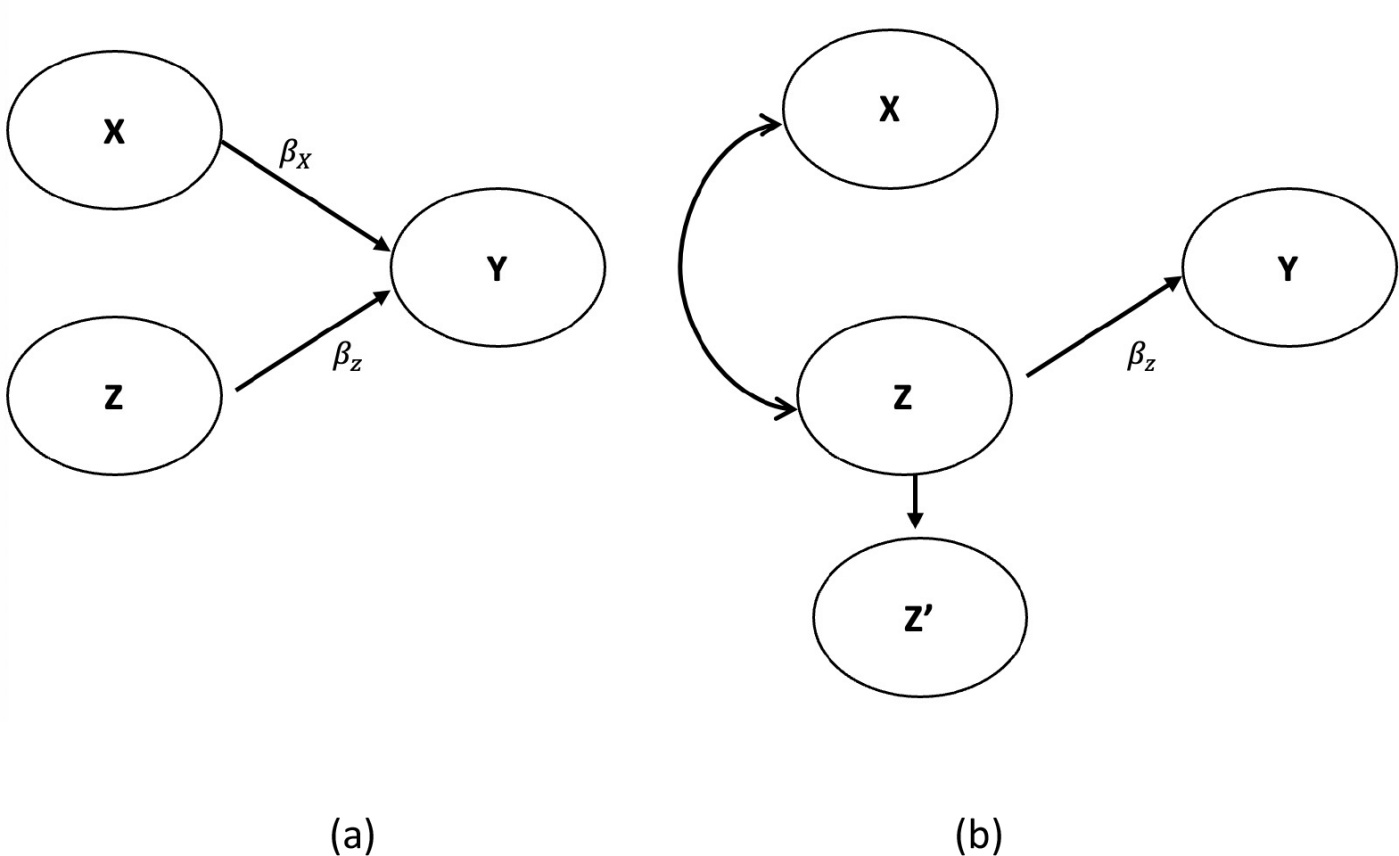
Agree (a) vs. disagree (b) with the interpretation of Misperception 5a. Demonstrates a nonlinear and non-monotonic association between body mass index (BMI) and mortality among U.S. adults aged 18–85 years old. This figure suggests that BMI ranging between 23–26 kg/m^2^ formed the nadir of the curve with the best outcome while persons with BMI levels below or above the nadir of the curve experienced increased mortality on average. Source: (Fontaine et al., 2003).

While the discussants’ conclusions may be entirely correct, as the philosophers of science say, the data are underdetermined by the hypotheses. That is, the data are consistent with the discussants’ interpretation, but that interpretation is not the only one with which the data are consistent. Therefore, the data do not definitively demonstrate the correctness of the discussants' tentative conclusions. There are alternative possibilities. In Figure 1, we show two DAGs consistent with the discussants’ conclusions. Yet they imply a completely different causal association between X and Y. Figure 1a is a simple DAG and agrees with the discussants’ conclusion. Figure 1b also agrees with the discussants’ conclusion, but X has no causal relationship with Y (no arrows). Yet, in some settings and some level of correlation between X and Z, X appears significant in a regression model with Z^’^ included in the model in lieu of Z.

First, there is tremendous collinearity between muscle mass and muscle strength. Given that almost all the pertinent human studies have non-experimental designs, the collinearity makes it especially difficult to determine whether there is cause and effect here and, if so, which of the two variables has a greater effect. With such strong multicollinearity between the strength and the mass measurements, any differential measurement error could make it appear that the more reliably measured variable had a greater causal effect over the less reliably measured variable, even if the opposite were true. Similarly, any differential nonlinearity of the effects of one of the two variables on the outcome relative to the others, if not effectively captured in the statistical modeling, could lead one variable to appear more strongly associated or effective than the other. In fact, the variable may just be more effectively modeled in this statistical procedure because of its greater linearity or greater conformity of its nonlinear pattern to the nonlinear model fit. We note that variance inflation factors are often used to diagnose multicollinearity in regressions.

Finally, even in linearly related sets of variables, the power to detect an association between a postulated cause and a postulated effect is highly dependent on the degree of variability in the causal factor in the population. If the variance were to be increased, the significance of the relationship would likely be accentuated. Thus, without an understanding of the measurement properties, the variability in the population, the variability which could exist in the population, and the causal structure among the variables, such analyses can only indicate hypotheses that are provisionally consistent with the data. Such analyses do not demonstrate that one variable does or does not definitively have a greater causal effect than the other or that one variable has a causal effect and the other variable does not. Note, regression coefficients within a model can be tested for equivalence in straightforward manners. Tests for non-trivial (non-zero) equivalence of some regression parameters can be done when it makes sense. In the linear regression model, testing for equivalence between parameters amounts to comparing the reduction in the sum of square error between a larger (in terms of number of parameters) model and a smaller model (with selected parameters constrained to be equal) relative to the large model sum of squares. The test then has an F distribution from which we can obtain the critical values and compute the p value (Neter et al., 1996).

#### How to avoid misperception 5a

Researchers should ensure that the variables to be adjusted for in the model are not too correlated to avoid multicollinearity issues. Variance inflation (VIF) tests available in most statistical software can be used to diagnose the presence of multicollinearity. Additionally, if measurement error or low covariate reliability is suspected, measurement error correction should be considered if possible.

### Misperception 5b. Controlling for the linear effect of a covariate is equivalent to controlling for the covariate

#### Why misperception 5b occurs

**Figure 2.**
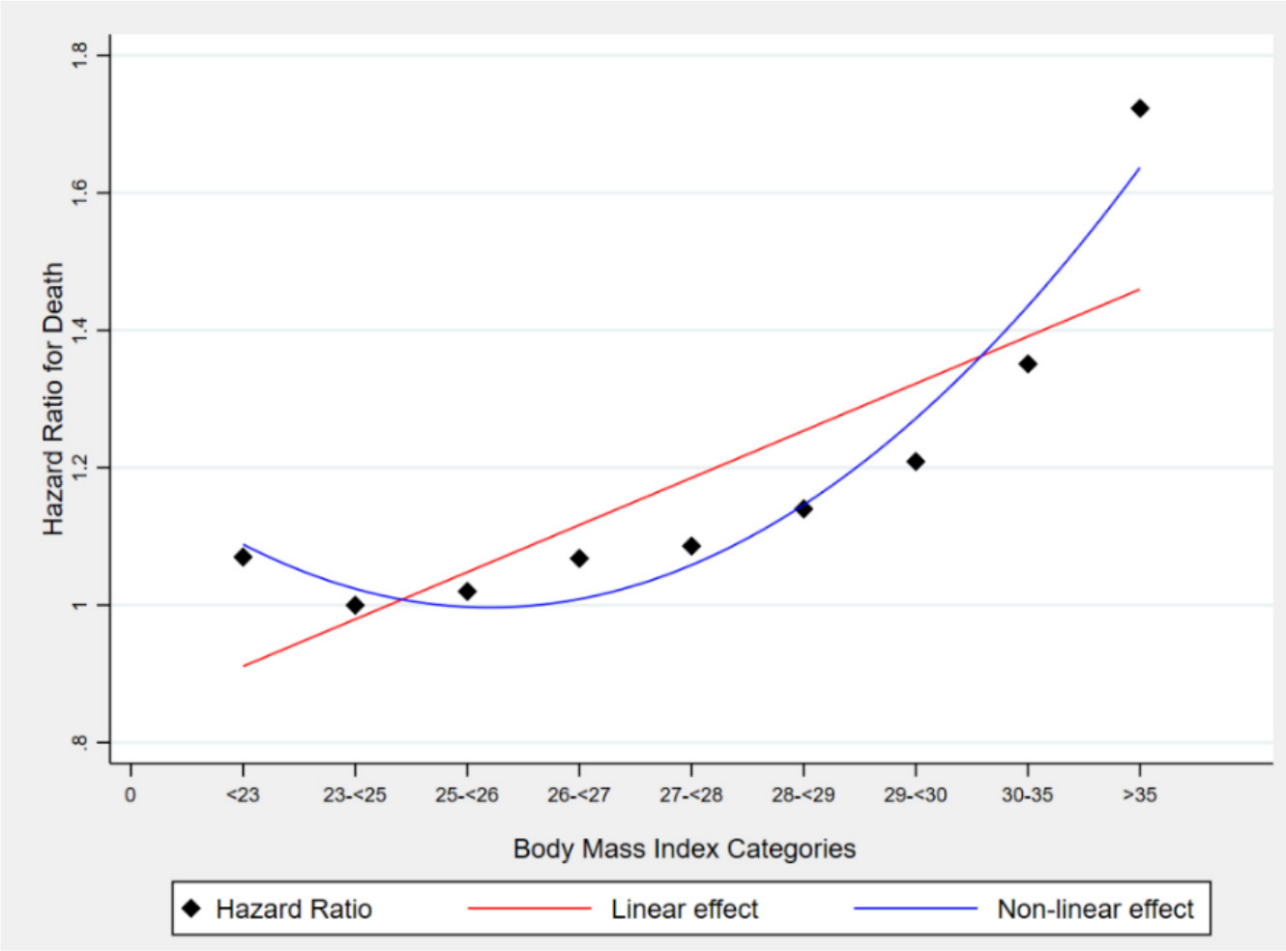
Association between body mass index and hazard ratio for death among U.S. adults aged 18–85 years old.

This assumption is not necessarily true because the relationships between some variables can be nonlinear. Thus, if one controls for only the linear term (which is typical) of a quantitative variable, say *Z*, as a PBC, then one does not effectively control for all the variance and potential bias induced by *Z*. The extent to which any residual bias in *Y* due to controlling *Z* only in its linear effects or association may be large or small depending on the degree of nonlinearity involved. In practice, much nonlinearity is monotonic. However, this is not true in all cases. For many risk factors such as body mass index (BMI), cholesterol, and nutrient intakes like sodium, there are often U-shaped (or more accurately concave upward) relationships in which persons with intermediate levels have the best outcomes and persons with covariate levels below or above the nadir of the curve have poorer outcomes, on average. An example of the nonlinear and non-monotonous relationship between BMI (the explanatory variable) and mortality (the outcome variable) is illustrated in Figure 2; Fontaine et al., 2003. In this example, mortality was treated as a time-to-event outcome modeled via survival analysis. This relationship has often been demonstrated to be U- or J-shaped (Fontaine et al., 2003; Flegal et al., 2007; Flegal et al., 2005; Pavela et al., 2022). Thus, when BMI is modeled linearly, the estimates will likely be potentially highly biased compared to when it is non-linearly modeled.

### How to avoid misperception 5b

It is important that one assesses for residual relationships (the relationships between nonlinear functions of *Z* and the model residuals after controlling for a linear function of *Z*) or chooses to allow for nonlinearity from the onset of the analysis. Nonlinearity can be accommodated through models that are nonlinear in the parameters (e.g. having parameters be exponents on the covariates) (Meloun and Militký, 2011; Andersen, 2009) or through use of techniques like the Box-Tidwell method transformations (Armstrong, 2017), splines (Schmidt et al., 2013; Oleszak, 2019), knotted regressions (Holmes and Mallick, 2003), categorical values (although see the next section for caveats around course categorization) (Reed Education, 2021), or good old-fashioned polynomials (Oleszak, 2019; Reed Education, 2021; Hastie et al., 2017) or in some cases factional polynomials (Sauerbrei et al., 2020; Binder et al., 2013; Royston and Altman, 1994; Royston and Sauerbrei, 2008).

## Misperception 6. One should check whether covariates are normally distributed and take corrective action if not

### Why misperception 6 occurs

This is not true. It is a common misperception that variables included in a parametric statistical model must be normally distributed. In fact, there is no requirement that any variable included in standard parametric regression or general linear models (Allison et al., 1993), either as a predictor or as a DV, be normally distributed. What is embedded in the *Gauss Markov Assumptions* (Berry, 1993), the assumptions of ordinary least-squares regression models (the models typically used in this journal), is that the residuals of the model be normally distributed. That is, the differences between the observed value of a DV for each subject and the predicted value of that DV from the model (and not any observed variable itself) are assumed to be normally distributed.

Moreover, this assumption about residuals applies only to the residuals of the DV. No assumption about the distribution of the predictor variables, covariates, or IV is made other than that they have finite mean and variance. Therefore, there is no need to assess the distributions of predictive variables, to take presumed corrective action if they are not normally distributed, or to suspect that the model is violated or biased if predictor variables are not normally distributed. One might be concerned with highly skewed or kurtotic covariates in that such distributions may contain extreme values, or outliers, that may serve as leverage points in the analysis, but that is a different issue. For an overview of outlier detection and the influence detection statistics best for managing concerns in this domain, see Fox, 2019.

### How to avoid misperception 6

This misperception can be avoided by recalling that in the regression model, the analysis is done conditional on the IVs (or covariates), which are assumed to be fixed. Thus, their distributions are irrelevant in the analysis. However, it is required that the residuals be uncorrelated with the IVs.

## Misperception 7. If the relation between a plausible confounder and the IV of interest is not *statistically significant*, the plausible confounder can be excluded with no concern for bias

In this misperception, the emphasis is on a relation that is *not statistically significant* instead of merely *not related*. This strategy is often implemented through stepwise regression techniques that are available in most statistical software. Statistical-significance-based criteria for including covariates can, if the predictor variable in question is actually a confounder (we rely on the word ‘confounder’ here for consistency with much of the scientific literature), lead to bias in both coefficient estimates and tests of statistical significance (Maldonado and Greenland, 1993; Greenland, 1989; Lee, 2014). As Greenland has pointed out, this “too often leads to deletion of important confounders (false negative decisions)” (Greenland, 2008). This is because the statistical-significance-based approach does not directly account for the actual degree of confounding produced by the variable in question.

### Why misperception 7 occurs

There could be confusion in understanding the nature of the question asked when selecting a variable for its confounding potential and the question asked in usual statistical significance testing (Dales and Ury, 1978). These two questions are fundamentally different. Even though a plausible confounder may not have a statistically significant association with the IV or the DV, or a statistically significant conditional association in the overall model, its actual association may still not be zero. That non-zero association in the population, even though not statistically significant in the sample, can still produce sufficient biases to allow false conclusions to occur at an inflated frequency. Additionally, a motivation for significance testing to select confounders may be to fit a more parsimonious final model in the large number of covariates and relatively modest sample size setting (VanderWeele, 2019). That is, false-positive decisions (i.e. selecting a harmless nonconfounder) are considered more deleterious than false-negative decisions (deleting a true confounder). It has been argued that the opposite applies: deleting a true confounder is more deleterious than including a harmless nonconfounder. The reason is that deleting a true confounder introduces bias and is only justified if the action is worth the precision gained. Whereas, including a harmless nonconfounder reduces precision, which is the price of protection against confounding (Greenland, 2008). We note that in not all circumstances is including a nonconfounder ‘harmless’ (Pearl, 2011).

### How to avoid misperception 7

Selection of confounders may be best when relying on substantive knowledge informing judgments of plausibility, the knowledge gained from previous studies in which similar research questions were examined, or a priori hypotheses and expectations for relationships among variables. If a variable is plausibly a confounder, it should be included in the model regardless of its statistical significance. As an additional approach, one can conduct the analysis with and without the confounder as a form of sensitivity analysis (VanderWeele and Ding, 2017; Rosenbaum, 2002) and report the results of both analyses. Such an approach is often referred to as the approach of the wise data analyst, who is willing to settle for, as Tukey defines, “an approximate answer to the right question, which is often vague, [rather] than an exact answer to the wrong question, which can always be made precise” (Tukey, 1962). We note that serious criticisms have been leveraged against the use of E-values in a sensitivity analysis as they tend to understate the residual confounding effect (Greenland, 2020; Sjölander and Greenland, 2022). However, attending to those critics is not within the scope of the current review.

## Misperception 8. Analyzing the residuals of an analysis in which a DV is regressed on the PBC is equivalent to including the PBC in an overall statistical model with the IV of interest

### Why misperception 8 occurs

This is untrue. As Maxwell pointed out several decades ago, the effects of analyzing residuals as opposed to including the PBC of interest in the model will depend on how those residuals are calculated (Maxwell et al., 1985). As Maxwell puts it, ANOVA on residuals is not ANCOVA. Maxwell shows that if the residuals are calculated separately for different levels of the IV, bias may accrue in one direction. In contrast, if residuals are calculated for the overall sample, bias may accrue in a different manner.

*Although this conceptualization of an equivalence between the two procedures* [ANOVA on residuals vs ANCOVA] *may be intuitively appealing, it is mathematically incorrect. If residuals are obtained from the pooled within-groups regression coefficient (b_w_), an analysis of variance on the residuals results in an inflated a-level. If the regression coefficient for the total sample combined into one group (b_T_) is used, ANOVA on the residuals yields an inappropriately conservative test. In either case, analysis of variance of residuals fails to provide a correct test, because the significance test in analysis of covariance requires consideration of both b_w_ and b_T_, unlike analysis of residuals* (Maxwell et al., 1985).

Notably, this procedure can introduce bias in the magnitude of the coefficients (effect sizes) characterizing the effects or associations of the IV of interest, and not just the test of statistical significance.

### How to avoid misperception 8

As Maxwell points out, there are ways to use residualization that do not permit these biases to occur. Hence, in some situations where models become so complex that residualizing for covariate effects beforehand makes the analysis that would otherwise be intractable tractable, this may be a reasonable approach. Nevertheless, additional concerns may emerge (Pain et al., 2018) and under ordinary circumstances, it is best to include PBCs in the model instead of residualizing for them first outside the model.

## Misperception 9. Excluding a covariate that is not associated with the outcome of interest does not affect the association of the IV with the outcome

### Why misperception 9 occurs

This is referred to as the suppressor effect. Adenovirus 36 (Ad36) infection provides an example of a suppressor effect. Although Ad36 increases adiposity, which is commonly linked to impaired glucoregulatory function and negative lipid profiles, Ad36 infection surprisingly leads to improved glucoregulatory function and serum lipid profiles (Akheruzzaman et al., 2019).

To illustrate the point, we set $\beta_{A}$ = 0.5, $\beta_{B}$ = –0.24, $\lambda_{A}$ = 0.8 and $\lambda_{B}$ = 0.6 implying zero-order correlation between the intake of fats of type B and *Y* would be zero. Yet, by controlling for fats of type B in the model, we would obtain an unbiased estimate of the effect of fats of type A on *Y* as $\beta_{A}$ , whereas if we did not control for fats of type B, we would mistakenly calculate the correlation between fats of type A and *Y* to be $\lambda_{A}\beta_{B}$ . This example demonstrates that failing to control for the suppressor variable, or the PBC that creates omitted variable bias, could result in a biased estimate of IV effects on the outcome, even when the suppressor variable has no correlation with the outcome. This disputes the premise that a covariate uncorrelated with the outcome cannot be biasing the results of an association test between another variable and the outcome as an indicator of a causal effect, thus undermining the original assumption. Whereas in the psychometrics literature, such patterns have commonly been termed *suppressor effects*, in a nutrition epidemiology paper they were referred to as *negative confounders* (Choi et al., 2008). We provide both theoretical and empirical justifications for these observations in Appendix A in the supplementary text file.

### How to avoid misperception 9

**Figure 3.**
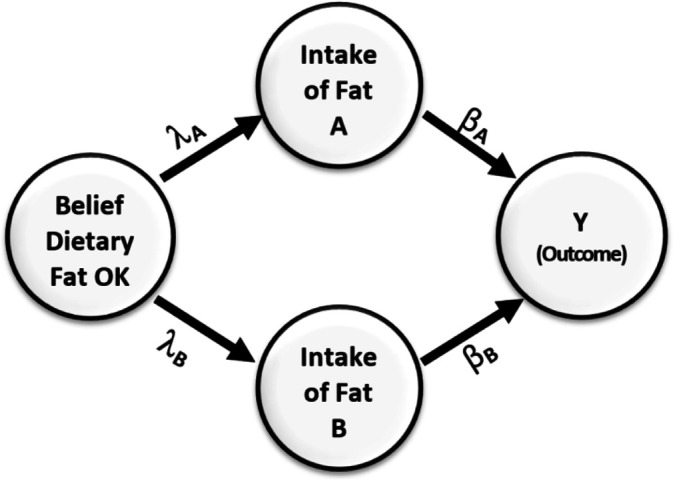
Causal relationships of health outcome, dietary fat consumption, and the belief that consumption of dietary fat is not dangerous. Direction of arrows represents causal directions and *λ_A_, λ_B_, β_A_*, and *β_B_* are structural coefficients.

This misperception is easily avoided if we refrain from only relying on marginal correlation to select covariates to include in the model and instead apply a backdoor criterion (Pearl and Mackenzie, 2018) to help decide which variables to adjust for and which to not adjust for. Provided that the directed acyclic diagram (DAG) in Figure 3 conforms to the true DAG, intake of fats B meets the backdoor criterion and must be adjusted for when estimating the effect of intake of fats, A on the outcome Y.

Figure 3 shows a simple causal model. On the left side of the figure is a variable representing an individual’s belief about the danger of dietary fat consumption. This belief affects their consumption of two types of fats, A and B. Fat type A is harmful and has a negative impact on health, while fat type B has a positive effect and improves health outcomes. The Greek letters on the paths indicate the causal effects in the model. Without loss of generality, we assume all variables have been standardized to have a variance of 1.0. From the rules of path diagrams (Alwin and Hauser, 1975; Bollen, 1987; Cheong and MacKinnon, 2012), we can calculate the correlations between *Y* and intake of fats of type B to be $\rho_{YB=\beta_{B}+\lambda_{B}\lambda_{A}\beta_{A}}$. This correlation is zero when $\lambda_{A}\lambda_{B}=\frac{-\beta_{B}}{\beta_{A}}$.

## Misperception 10. If a plausible confounding variable is one that has a bivariate unadjusted correlation of zero with the IV, then it does not create bias in the association of the IV with the outcome

### Why misperception 10 occurs

This misperception is based on the same premises as stated above but manifests differently. Let us replace ‘confounding variable’ with ‘PBC,’ which we defined earlier. For Misperception 10, the presumption is that a PBC, if not properly included and controlled for in the design or analysis, will only bias the extent to which the association between the IV and the DV represents the cause or effect of the IV on the DV if the PBC is related to *both* the IV and the DV.

**Figure 4.**
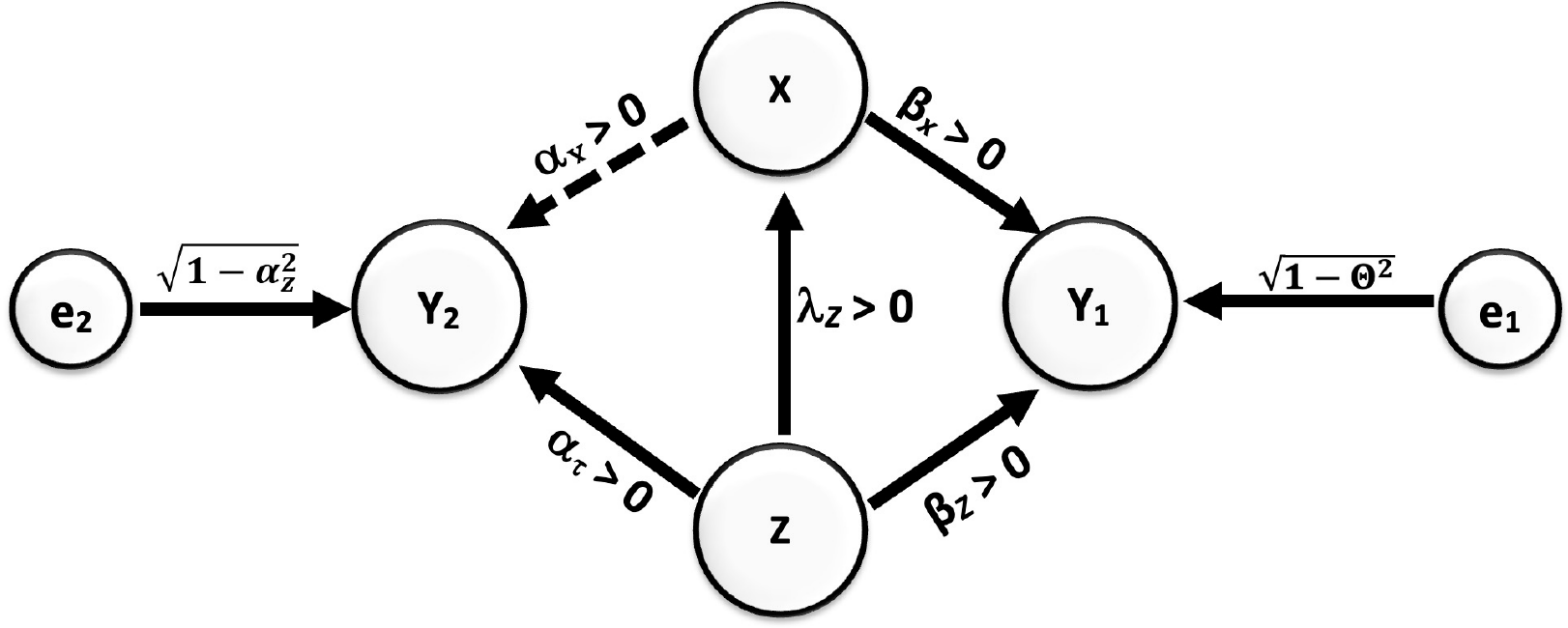
Causal relationships of outcome, covariate, and potentially biasing covariate (PBC). Direction of arrows represents causal directions and *λ_z_,* α*_z_,* α*_x_, β_z_*, and *βx* are structural coefficients. The error terms e1 and e2 have variances chosen so Y1 and Y2 have variances 1 (see the Appendices for more details).

Under those assumptions, if we consider a PBC and find that it is one that has a bivariate unadjusted correlation of zero with the IV, then it cannot be creating bias. Yet, this is not true. Multiple circumstances could produce a pattern of results in which a biasing variable has a correlation of zero, as well as no nonlinear association with the outcome, and yet creates a bias if not properly accommodated by design or analysis. Moreover, there may be circumstances in which statistically adjusting for a variable does not reduce the bias even though in other circumstances such adjustment would. Consider the causal model depicted in Figure 4, which follows the same notational conventions as Figure 3.

In this case, both *X* and *Z* have a causal effect on *Y*_1_. *Y*_1_ can then be referred to as a ‘collider’ (see Glossary). It is well established that conditioning on a collider will alter the association between joint causes of it. Most often, collider bias is discussed in terms of creating associations. For example, in the figure shown here, if *Z* and *X* were not correlated, but both caused increases in *Y*_1_, then conditioning on (i.e. ‘controlling for’) *Y*_1_ would create a positive or negative correlation between *X* and *Z*. However, as Munafò et al., 2018 explain, collider bias need not simply create associations, but can also reduce or eliminate associations: “Selection can induce collider bias… which can lead to biased observational… associations. This bias can be towards or away from any true association, and can distort a true association or a true lack of association.”

In Figure 4 Appp, there is an association between *X* and *Z*, and *Z* would be the PBC (confounding variable in conventional terminology) of the relationship between *X* and *Y*_1_ and *Y*_2_. But, if we set up the coefficients to have certain values, selecting on *Y*_1_ (for example, by studying only people with diagnosed hypertension defined as a systolic blood pressure greater than 140 mm Hg) could actually drive the positive association between *X* and *Z* to zero. Specifically, for these coefficient values [β_x_ = 0.1857, , β_z_ = 0.8175, λ_z_ = 0.4, α_x_ = 0.0, α_x_ = 0.6], if all variables were normally distributed (in the derivation in Appendix 2, we assume that all variables have a joint multivariate normal distribution. Whether this applies to cases in which the data are not multivariate normal is not something we have proven one way or another). with mean zero and standard deviation 1 (this would be after standardization of the variables), then using a cutoff of approximately 1.8276 standard deviations above the mean of *Y*_1_ would cause the correlation in that subsample between *X* and *Z* to be zero (Arnold and Beaver, 2000; Azzalini and Capitanio, 2013).

Furthermore, let us assume that all the relations in this hypothetical circumstance are linear. This can include linear relationships of zero, but no nonlinear or curved relationships. Here, when we control for the PBC Z in the selected sample of persons with hypertension, it will have no effect on the estimated slope of the regression of *Y*_2_ on *X*. The collider bias has altered the association between *Z* and *X* such that controlling for *Z* in a conventional statistical model, i.e., an ordinary least-squares linear regression, no longer removes the bias. And yet, the bias is there. We justify this through mathematical arguments along with a small simulation to elucidate the manifestation of this misperception in Appendix 2.

More sophisticated models involving missing data approaches and other approaches could also be brought to bear (Groenwold et al., 2012; Yang et al., 2019; Greenwood et al., 2006), but this simple example shows that just because a PBC has no association with a postulated IV (i.e. cause), this does not mean that the variable cannot be creating bias (confounding) in the estimated relationship between the postulated IV and the postulated result or outcome. In the end, as Pedhazur put it, quoting Fisher, “If…we choose a group of social phenomena with no antecedent knowledge of the causation or the absence of causation among them, then the calculation of correlation coefficients, total or partial, will not advance us a step towards evaluating the importance of the causes at work…In no case, however, can we judge whether or not it is profitable to eliminate a certain variate unless we know, or are willing to assume, a qualitative scheme of causation” (Fisher, 1970).

In the end, there is no substitute for either randomization or, at a minimum, informed argument and assumptions about the causal structure among the variables. No simple statistical rule will allow one to decide whether a covariate or its exclusion is or is not creating bias.

### How to avoid misperception 10

Selecting or conditioning on a collider can bias estimated effects in unforeseeable ways. Given a causal DAG, the use of the backdoor criterion can help the analyst identify variables that can safely be adjusted for and those that can bias (confound) the effect estimate of interest. In Figure 4, for example, Y_1_ does not meet the backdoor criterion from Y_2_ to X, and adjusting for it or selecting on it will bias the estimate of the effect estimate.

## Misperception 11. The method used to control for a covariate can be assumed to have been chosen appropriately and other methods would not, on average, produce substantially different results

This is, in essence, a statement of the unbiasedness of an analytic approach. By this we mean that the method of controlling for the covariate is not chosen, intentionally or unintentionally, to achieve a particular study finding, and that the answer obtained does not deviate from the answer one would get if one optimally controlled for the covariate. By ‘optimally controlled,’ we mean using a method that would eliminate or reduce to the greatest extent possible any effects of not controlling for the covariate and that is commensurate with the stated goals of the analysis (which is more important than the interests of the investigator).

Unfortunately, we have substantial evidence from many sources that many investigators instead choose analytical approaches, including the treatment of covariates, that serve their interests (e.g. Head et al., 2015; Wicherts et al., 2016; Bruns and Ioannidis, 2016). Conventionally, this is termed ‘p-hacking’ (Simonsohn et al., 2014), ‘investigator degrees of freedom’ (Simmons et al., 2011), ‘taking the garden of forking paths’ (Gelman and Loken, 2013), and so on. If such methods are used, that is, if investigators try multiple ways of controlling for which, how many, or form of covariates until they select the one that produces the results most commensurate with those they wish for, the results will most certainly be biased (Sturman et al., 2022; Kavvoura et al., 2007; Banks et al., 2016; O’Boyle et al., 2017; Simmons et al., 2011; Christensen et al., 2021; Stefan and Schönbrodt, 2022; Austin and Brunner, 2004).

### Why misperception 11 occurs

To our knowledge, surveys do not exist describing the extent to which authors are aware of the consequences of intentionally choosing and reporting models that control for covariates to obtain a certain result. Some evidence exists, however, that suggests authors do sometimes intentionally select covariates to achieve statistical significance, such as a survey by Banks et al. of active management researchers (Banks et al., 2016). O’Boyle et al. observed changes in how control variables were used in journal articles compared with dissertations of the same work, with the final publications reporting more statistically significant findings than the dissertations (O’Boyle et al., 2017). Research on the motivations of these practices may help to focus preventive interventions.

### How to avoid misperception 11

This concern with *P*-hacking is one of the major impetuses behind those in our field encouraging investigators in observational studies to preregister their analyses (; Dal Ré et al., 2014). Many steps in the model-building process could consciously or unconsciously influence the probability of type I error, from the conceptualization of the research question (e.g. the quality of prior literature review, discussions with collaborators and colleagues that shape modeling choices), to any prior or exploratory analysis using that dataset, or to the numerous analytical decisions in selecting covariates, selecting their forms, accounting for missing data, and so on. Future theoretical and empirical modeling is needed to inform which decisions have the least likelihood of producing biased findings.

However, that is not to say that investigators should not limit their flexibility in each of these steps, engage in exploratory analyses, or change their minds after the fact—or that we do not do that ourselves. But this should be disclosed so that the reader can make an informed decision about what the data and results mean. Within our group, we often say colloquially, we are going to analyze the heck out of these data and try many models, but then we are then going to disclose this to the reader. Indeed, transparency is often lacking for how the inclusion or form of adjustment is determined in observational research (Lenz and Sahn, 2021). In situations where authors want to explore how covariate selection flexibility may affect results, so-called multiverse-style methods (Steegen et al., 2016) (also called vibration of effects Patel et al., 2015) or specification curve analysis (Simonsohn et al., 2020) can be used, although careful thought is needed to ensure such analyses do not also produce misleading conclusions (Del Giudice and Gangestad, 2021).

## Misperception 12. p values derived from implementing statistical methods incorporating covariates mean exactly what they appear to mean and can be interpreted at face value

### Why misperception 12 occurs

This is not necessarily true. An article from many years ago discusses the problem of a reproducible ‘Six Sigma’ finding from physics (Linderman et al., 2003). A Six Sigma finding is simply a finding whose test statistic is six or more standard deviations from the expectation under the null hypothesis. Six Sigma findings should be indescribably rare based on known probability theory (Actually, they are exactly describably rare and should occur, under the null hypothesis, 10e-10 proportion of the time.). However, it seems that all too often, Six Sigma findings, even in what might be seen as a mature science like physics, are regularly overturned (Harry and Schroeder, 2005; Daniels, 2001). Why is this? There are likely multiple reasons, but one is plausible that the assumptions made about the measurement properties of the data, the distributions of the data, and the performance of the test statistics under violations of their pure assumptions were not fully understood or met (Hanin, 2021). This issue involving violations of assumptions of statistical methods (Greenland et al., 2016) may be especially important when dealing with unusually small alpha levels (i.e. significance levels) (Bangalore et al., 2009). This is because a test statistic that is highly robust to even modest or large violations of some assumptions at higher alpha levels such as 0.05 may be highly sensitive to even small violations of assumptions at much smaller alpha levels, such as those used with Six Sigma results in physics. Another example is with the use of multiple testing ‘corrections’ in certain areas like genetic epidemiology with genome-wide association testing in nutrition and obesity research, where significance levels of 10e-8 are commonly used and p values far, far lower than that are not infrequently reported.

### How to avoid misperception 12

In short, robustness at one significance level does not necessarily imply robustness at a different significance level. Independent replication not only takes into account purely stochastic sources of error but also potentially allows one to detect the inadvertent biasing effects of other unknown and unspecifiable factors beyond stochastic variation.

## Discussion

We have discussed 12 issues involving the use of covariates. Although our description of each misperception is mostly done in a linear model setting, we note that these issues also remain in the nonlinear model. We hope that our attention to these issues will help readers better understand how to most effectively control for potential biases, without inducing further biases, by choosing how and when to include certain covariates in the design and analysis of their studies. We hope the list is helpful, but we wish to note several things. First, the list of issues we provide is not exhaustive. No single source, that we are aware of, will necessarily discuss them all, but some useful references exist (Cinelli et al., 2020; Gelman et al., 2020; Ding and Miratrix, 2015). Second, by pointing out a particular analytical approach or solution, we do not mean to imply that these are the only analytic approaches or solutions available today or that will exist in the future. For example, we have not discussed the Bayesian approach much. Bayesian approaches differ from their non-Bayesian counterparts in that the researcher first posits a model describing how observable and unobservable quantities are interrelated, which is often done via a graph. Many of the misconceptions detailed here are related to covariate selection bias and omitted or missing covariates bias, which can be corrected for in a Bayesian analysis provided it is known how the unobserved variables are related to other model terms (see (McElreath, 2020) for an accessible and concise introduction to Bayesian analysis and its computation aspects). Third, most of the misconceptions discussed here and ways to avoid them have a direct connection with causal inference. Namely, assuming a DAG depicting the data-generating process, we can use the front-door or front-door criterion derived from the do-calculus framework of Pearl and Mackenzie, 2018; Pearl et al., 2016. Determination of the adjusting set in a DAG can sometimes be challenging, especially in larger DAGs. The freely available web application dagitty (https://www.dagitty.net/) allows users to specify their DAGs and the application provides the set of controlling variables (Textor et al., 2016).

We encourage readers to seek the advice of professional statisticians in designing and analyzing studies around these issues. Furthermore, it is important to recognize that no one statistical approach to the use or nonuse of any particular covariate or set of covariates in observational research will guarantee that one will obtain the ‘right’ answer or an unbiased estimate of some parameter without demanding assumptions. There is no substitute for the gold standard of experimentation: strictly supervised double-blind interventional experiments with random selection and random assignment. This was aptly illustrated in a study by Ejima et al., 2016. This does not mean that one should not try to estimate associations or causal effects in observational research. Indeed, as Hernán effectively argues (Hernán, 2018), we should not be afraid of causation. When we do much observational research, we are interested in estimating causal effects. But we must be honest: what we are actually estimating is associations, and we can then discuss the extent to which those estimates of associations may represent causal effects. Our ability to rule out competing explanations for the associations observed, other than causal effects, strengthens the argument that the associations may represent causal effects, and that is where the wise use of covariates comes in. But such arguments used with covariates do not demonstrate causal effects, they merely make more or less plausible in the eyes of the beholder that an association does or does not represent causation. In making such arguments, as cogently noted on the value of epistemic humility and how to truly enact it, “Intellectual humility requires more than cursory statements about these limitations; it requires taking them seriously and limiting our conclusions accordingly” (Hoekstra and Vazire, 2021). That is, consideration of arguments about the plausibility of causation from association should not be given in such a way as to convince the reader, but rather to truly give a fair and balanced consideration of the notion that an association does or does not represent a particular causal effect. As Francis Bacon famously said, “Read not to contradict and confute; nor to believe and take for granted; nor to find talk and discourse; but to weigh and consider” (Bacon, 2022).

### Data availability

All data generated or analyzed during this study are included in the manuscript and supplementary files; R studio software used for the description and illustration of misperception 5 a, misperception 9 and misperception 10 are publicly available on GitHub.

## Figures and Tables

**Appendix 1—figure 1.**
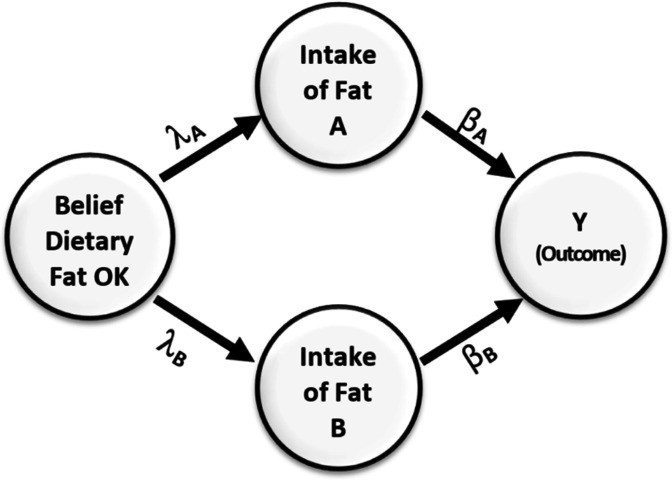
Causal relationships of health outcome, dietary fat consumption, and the belief that consumption of dietary fat is not dangerous. Direction of arrows represents causal directions and 𝜆_*A*_, 𝜆_*B*_, 𝛽_*A*_, and 𝛽_*B*_ are structural coefficients.

**Appendix 2—figure 1.**
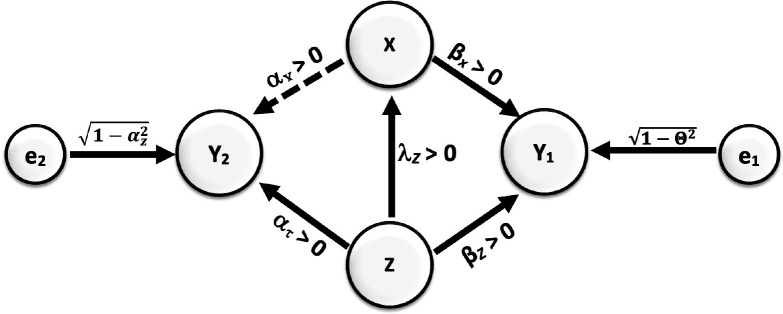
Causal relationships of outcome, covariate, and confounding. Direction of arrows represents causal directions and 𝜆_*z*_, 𝛼_*z*_, 𝛼_*x*_*,* 𝛽_*z*_, and 𝛽_*x*_ are structural coefficients.

**Appendix 2—figure 2.**
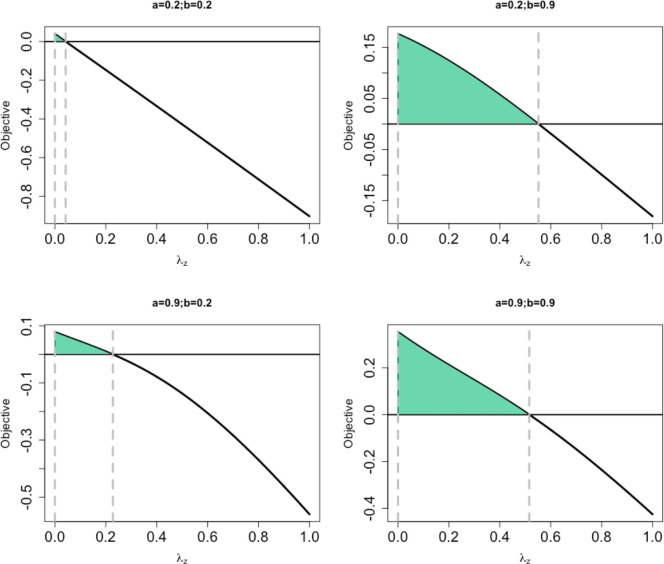
Possible values of $\lambda_{z}$ based on each choice of the pairs of a, b. The area shaded in green denotes the area for which a $\lambda_{z}$ value has a value τ that makes Equation 13 equal zero.

## Appendix 1

### Misperception 9. Excluding a covariate that is not associated with the outcome of interest does not affect the association of the IV with the outcome

Consider a simple causal model depicted in Appendix 1—figure 1 (Figure 2 in the main text). At the left side of Appendix 1—figure 1, we have a variable that is the degree of one’s belief that dietary fat consumption is not dangerous or, conceived alternatively, one minus the strength of belief that dietary fat consumption is dangerous or should be avoided. Suppose this variable relates to dietary consumption of two kinds of dietary fats, and , where dietary fat of type A decreases some health outcome of interest (i.e. is harmful). In contrast, dietary fat of type B decreases the negative health outcome (i.e. is helpful).

We can use the following linear model to describe the causal effects in Appendix 1—figure 1:

(1) $$M_{F}:Y=\beta_{0}+\beta_{A}X_{A}+\beta_{B}X_{B}+\epsilon$$

where Y is the response variable, $X_{A}$ and $X_{B}$ are IVs representing the fat consumptions of dietary fat types *A* and *B*, respectively, and ε is an independent error term with the variance $\sigma_{\varepsilon }^{2}$ . Of the two covariates, we suppose *X*_*A*_ is the exposure of interest that is correlated with *Y* and *X_B_* is a confounding variable that is correlated with *Y*, resulting in the correlations $\rho (X_{A},Y)\neq 0$, $\rho (X_{B},Y)=0$, respectively. Following the causal diagram in Appendix 1—figure 1, we generate *X_A_* and *X_B_* from a latent variable Z, where

$$\begin{array}{c} X_{A}=\lambda_{A}Z+\eta \\ X_{B}=\lambda_{B}Z+\gamma \end{array}$$

where $\lambda_{A}\neq o,\lambda_{B}\neq 0$ and *η* and *γ* are independent error terms with variances $\sigma_{\eta }^{2}$ and $\sigma_{\gamma }^{2}$, respectively. Without loss of generality, we assume that variables *X_A_*, *X_B_*, and *Z* have been standardized to unit variance, and the additional regression parameters are chosen so that the *Y* also has unit variance. This then implies the causal effects $\rho (Y,X_{A})=\beta_{A}+\beta_{A}\lambda_{A},\lambda_{B}$, $\rho (Y,X_{B})=\beta_{B}+\beta_{A}\lambda_{A},\lambda_{B}$, and $\rho (X_{A},X_{B})=\lambda_{A},\lambda_{B}$_*.*_

Consider the following reduced model where the confounding variable, *X_B_*, is excluded from the full model (1):

(2) $$M_{R}:Y=\beta_{0}+\beta_{A}X_{A}+\varepsilon$$

and $\varepsilon \equiv \beta_{B}X_{B}+\varepsilon$. The least-squares estimate for *β*_*A*_ under the reduced model (2) is

$$\begin{array}{c} {\hat{\beta }}_{A}=\frac{Cov(Y,X_{A})}{Var(X_{A})}\\ =Cov(\beta_{0}+\beta_{A}X_{A}+\epsilon^{*},X_{A})\\ =\beta_{A}+Cov(\epsilon^{*},X_{A})\\ =\beta_{A}+Cov(\beta_{B}X_{B}+\epsilon ,X_{A})\\ =\beta_{A}+\beta_{B}Cov(X_{B},X_{A})\\ =\beta_{A}+\beta_{B}+\lambda_{A}\lambda_{B} \end{array}$$

Under the assumption that $\rho (Y,X_{B})=0$, we have $\beta_{B}=-\beta_{A}\lambda_{A}\lambda_{B}$. Plugging this into equation ${\hat{\beta }}_{A}$, we have

$${\hat{\beta }}_{A}=\beta_{A}+\beta_{A}\lambda_{A}\lambda_{B}=\beta_{A}(1-\lambda_{A}^{2}+\lambda_{B}^{2})\neq \beta_{A}$$

Since $\lambda_{A}\neq 0$, $\lambda_{B}\neq 0$. The above derivation demonstrates that omitted variable bias cannot be avoided under the imposed assumption in the causal model of Appendix 1—figure 1. However, those requirements contradict the imposed assumption in the causal model of Appendix 1—figure 1 indicating that the omitted variable bias cannot be avoided.

Despite the theoretical justification, we conducted simulation studies to illustrate our points. To generate simulated data under the imposed assumptions, we select regression parameters following the restrictions:

(3) $$\beta_{A}+\beta_{B}\lambda_{A}\lambda_{B}\neq 0$$

(4) $$\beta_{B}+\beta_{A}\lambda_{A}\lambda_{B}=0$$

(5) $$\lambda_{A}^{2}+\sigma_{\eta }^{2}=1$$

(6) $$\lambda_{B}^{2}+\sigma_{\gamma }^{2}=1$$

(7) $$\beta_{A}^{2}+\beta_{B}^{2}+\sigma_{\epsilon }^{2}+2\beta_{A}\beta_{B}\lambda_{A}\lambda_{B}=1$$

where restrictions (5), (6), and (7) are required to have $Var(X_{A})=1$, $Var(X_{B})=1$, and Var(Y)=1, respectively. Plugging (5) and (6) into (3) yields $\sigma_{\gamma }^{2}+\sigma_{\eta }^{2}-\sigma_{\gamma }^{2}\sigma_{\eta }^{2}\neq 0$. We consider simulation settings based on the parameter specifications presented in Appendix 1—table 1, where variables Z,ε,η, and γ were generated from independent normal distributions with zero means. For all scenarios considered, the empirical Pearson’s correlations between Y and $X_{B}$ are close to zero. With the simulated data, we examined the bias of least-squares estimator for $\beta_{A}$ under the full model of (1) and the reduced model (2). With 10,000 replications and three levels of sample sizes nϵ{500,1000,2000}, the summary of bias is presented in Appendix 1—table 2. As expected, the bias of $\beta_{A}$ is virtually zero when controlling for $X_{B}$ in the full model. On the contrary, failing to control for $X_{B}$ in the model, one would mistakenly estimate the causal effect between $X_{A}$ and Y resulting in a bias that agrees closely to $\beta_{A}\lambda_{A}\lambda_{B}$*_._* Our simulation results confirm that excluding confounding variables from the model could bias the coefficient estimates, hence introducing omitted variable biases. In addition, our results dispute the premise that a covariate that is uncorrelated with the outcome cannot be biasing the results of an association test between another variable and the outcome as an indicator of a causal effect and disputes the premise we began with. Whereas in the psychometrics literature such patterns have usually been termed *suppressor effects*, in a nutrition epidemiology paper they were referred to as *negative confounders* (Choi et al., 2008).

**Appendix 1—table 1.** Parameters used to generate simulated data for the simulation studies under Misperception 9.

Scenario	β_A_	*β*_*B*_	*λ*_*A*_	*λ*_*B*_	$\sigma_{\epsilon }^{2}$	$\sigma_{\eta }^{2}$	$\sigma_{\gamma }^{2}$
I	–0.4	0.3	$\sqrt{3}/2$	$\sqrt{3}/2$	0.93	0.25	0.25
II	0.4	–0.3	$\sqrt{3}/2$	$\sqrt{3}/2$	0.93	0.25	0.25
III	–0.5	0.24	0.8	0.6	0.8076	0.36	0.64
IV	0.5	–0.24	0.8	0.6	0.8076	0.36	0.64

**Appendix 1—table 2.** Summary of bias when fitting the full model (𝑀_𝐹_) and the reduced model (*M*_*R*_). The bias is defined as $\hat{\beta }-\beta_{A}$, where $\hat{\beta }$ is the least-squares estimate under the corresponding model.

Scenario	n=500		n=1000		n=2000	
	*M*_*F*_	*M*_*R*_	*M*_*F*_	*M*_*R*_	*M_F_*	*M*_*R*_
I	–0.0007	0.2248	0.0001	0.2251	–0.0001	0.2249
II	0.0005	–0.2249	0.0003	–0.2249	0.0002	–0.2248
III	–0.0001	0.24	0.0003	0.2405	–0.0003	0.2399
IV	0.0004	–0.2396	–0.0002	–0.24	–0.0005	–0.2402

## Appendix 2

### Misperception 10. If a plausible confounding variable is unrelated to the IV, then it does not create bias in the association of the IV with the outcome

To illustrate this misconception, let’s consider the causal diagram shown in Appendix 2—figure 1.

$$\Theta^{2}=\beta_{z}^{2}+\beta_{x}^{2}\lambda_{z}^{2}+\beta_{x}^{2}+2\beta_{z}\beta_{x}\lambda_{z}$$

where

Data-generating model:

Let us consider the setting we had in the description of Misconception 9 in Appendix 1. Furthermore, $Y_{1}$ and $Y_{2}$ are the outcomes or DVs, *X* is the covariate of primary interest, and *Z* is the confounder in the casual association of the covariate with each response. We have the following model:

$$Y_{1}=\beta_{x}X+\beta_{z}Z+\epsilon_{1}$$

$$Y_{2}=\alpha_{x}X+\alpha_{z}Z+\epsilon_{2}$$

$$X=\lambda_{z}Z+\epsilon_{x}$$

We further assume that *Z* has a Gaussian distribution with mean 0 and variance 1; $\epsilon_{x}$ has a normal distribution with mean zero and variance $\sigma_{x}^{2}$ ; $e_{1}$ has a normal distribution with mean zero and variance $\sigma_{\epsilon ,1}^{2}$ ; and $e_{2}$ has a normal distribution with mean zero and variance $\sigma_{\epsilon ,2}^{2}$ . Without loss of generality, we select the variance term so that the outcomes $Y_{1}$ and $Y_{2}$ and the exposure *X* and the confounder *Z* all have unit variance. The joint distribution of (*Z*, *X*, $Y_{2},Y_{1}$) is a multivariate normal distribution with mean zero vector and the correlation matrix provided in Appendix 2—table 1.

Where $\sigma_{\epsilon ,1}^{2}=1-\left(\beta_{z}^{2}+\beta_{x}^{2}\lambda_{z}^{2}+2\beta_{z}\beta_{x}\lambda_{z}+\beta_{x}^{2}\right);\sigma_{\epsilon ,2}^{2}=1-\left(\alpha_{z}^{2}+\alpha_{x}^{2}\lambda_{z}^{2}+2\alpha_{z}\alpha_{x}\lambda_{z}+\alpha_{x}^{2}\right)$ ; $\sigma_{x}^{2}=1-\lambda_{z}^{2}$ . Thus, $Var\left(Y_{1}\right)=Var\left(Y_{2}\right)=1$. This clearly implies the following constraints on the parameters $0<\beta_{z}<\sqrt{1-\beta_{x}^{2}}-\lambda_{z}\beta_{x}$, $0<\alpha_{z}<\sqrt{1-\alpha_{x}^{2}}-\lambda_{x}\alpha_{x}$.

(8) $$0\leq \beta_{z}\leq \sqrt{1-\beta_{x}^{2}}-\lambda_{z}\beta_{x}$$

(9) $$0\leq \beta_{x}^{2}\leq 1/\left(1+\lambda_{z}^{2}\right)$$

(10) $$0\leq \alpha_{z}\leq \sqrt{1-\alpha_{x}^{2}}-\lambda_{x}\alpha_{x}$$

(11) $$0\leq \alpha_{x}^{2}\leq 1/\left(1+\lambda_{z}^{2}\right)$$

**Appendix 2—table 1.** The correlation matrix among *Z*, *X*, *Y*_2_, and *Y*_1_ without selecting on *Y*_1_.

Σ	Z	X	Y_2_	Y_1_
Z	1	$\lambda_{z}$	$\alpha_{z}+\alpha_{x}\lambda_{z}$	$\beta_{z}+\beta_{x}\lambda_{z}$
X	$\lambda_{z}$	1	$\alpha_{x}+\alpha_{z}\lambda_{z}$	$\beta_{x}+\beta_{z}\lambda_{z}$
Y_2_	$\alpha_{z}+\alpha_{x}\lambda_{z}$	$\alpha_{x}+\alpha_{z}\lambda_{z}$	1	$\left(\alpha_{z}+\alpha_{x}\lambda_{z})\;\beta_{z}+\beta_{x}(\alpha_{x}+\alpha_{z}\lambda_{z}\right)$
Y_1_	$\beta_{z}+\beta_{x}\lambda_{z}$	$\beta_{x}+\beta_{z}\lambda_{z}$	$\left(\alpha_{z}+\alpha_{x}\lambda_{z})\beta_{z}+\beta_{x}(\alpha_{x}+\alpha_{z}\lambda_{z}\right)$	1

Suppose we restrict the sample to values of $Y_{1}>E\left(Y_{1}\right)+\tau \sigma_{Y_{1}}$ and $var\left(Y_{1}\right)=1$. This will ultimately perturb the joint distribution of $\left(Z,X,Y_{2}\right)$ . We can analytically derive the joint distribution of $\left(Z,X,Y_{2}\right)\vee Y_{1}>\tau$. Using results from (2), the joint distribution of $\left(Z,X,Y_{2}\right)\vee Y_{1}>\tau$ is an extended multivariate skew-normal. Namely, the density of the vector is

$$f\left(\upsilon |Y_{1}>\tau \right)=\frac{\Phi \left(\alpha^{T}\upsilon +\alpha_{0}\right)}{\Phi \left(-\tau \right)}\phi (\upsilon^{T}\sum_{12}^{-1}\upsilon )$$

Where $\phi \left(.\right)$ and $\Phi \left(.\right)$ denote the density function and the cumulative density function of the normal distribution. After selecting on values of $Y_{1}>\tau$

(12) $$E\left(\upsilon \right)=\frac{\phi \left(-\tau \right)}{\Phi \left(-\tau \right)}\rho$$

(13) $$\sigma_{ij}^{2}=\sigma_{12,ij}+\frac{\phi \left(-\tau \right)}{\Phi \left(-\tau \right)}\left(\tau -\frac{\phi \left(-\tau \right)}{\Phi \left(-\tau \right)}\right)\rho_{i}\rho_{j}$$

where

$$\rho =\left\{\beta_{z}+\beta_{x}\lambda_{z},\beta_{x}+\beta_{z}\lambda_{z},\beta_{z}+\beta_{x}(\alpha_{x}+\alpha_{z}\lambda_{z})\right\}$$

and $\rho_{i}$ is the ith element of ρ and $\sigma_{ij}^{2}$ is the entry of the matrix in Appendix 2—table 1 at row i and column j. To find τ so that $\sigma_{12}^{2}=cor\left(X,Z|Y_{1}>\tau \right)=0$, we need to solve the following equation for τ

$$\lambda_{z}+\frac{\phi \left(-\tau \right)}{\Phi \left(-\tau \right)}\left(\tau -\frac{\phi \left(-\tau \right)}{\Phi \left(-\tau \right)}\right)\left(\beta_{z}+\lambda_{z}\beta_{x}\right)\left(\beta_{x}+\lambda \beta_{z}\right)=0$$

Since the quantity on the right-hand side is non-negative and takes on a value between 0 and 1, then for any choices of the triplet $\left(\lambda_{z},\beta_{x},\beta_{z}\right)$ , where $\lambda_{z}<\left(\beta_{z}+\beta_{x}\lambda_{z}\right)\left(\beta_{x}+\beta_{x}\lambda_{z}\right)$ , we can find a τ so that $corr\left(X,Z\right)=0$ based on the data for which $Y_{1}>\tau .$ Let’s further assume that for a given $\lambda_{x},\beta_{x}=\frac{a}{\sqrt{1+\lambda_{x}^{2}}}\wedge \beta_{z}=b\left(\frac{\sqrt{1+\lambda_{x}^{2}+a^{2}}-\lambda_{x}a}{\sqrt{1+\lambda_{x}^{2}}}\right)=\frac{b}{a}\beta_{x}\left(\sqrt{1+\lambda_{x}^{2}+a^{2}}-\lambda_{x}a\right),$ for fixed $0<\lambda_{z}<1,\wedge 0\leq a,b\leq 1.$ Thus for any pair (a,b)with0≤a,b<1, we have a set of possible values of $\lambda_{z}$ that satisfies all the constraints enumerated above. In the setting, $\beta_{x}\wedge \beta_{z}$ involve the constants $a,b,\wedge \lambda_{z}$ in a nonlinear fashion. We rely on numerical approaches to identify values of $\lambda_{z}$ consistent with the values of a∧b. We illustrate this with the case where a,b∈{0.2,0.9}, which results in four possible pairs $\left(0.2,0.2\right)$ , $\left(0.2,0.9\right)$ , $\left(0.9,0.2\right)$ , and $\left(0.9,0.9\right).$ In Appendix 2—figure 2, we show the set of possible values of $\lambda_{z}$ , combined with the value of $\beta_{x},\beta_{z}$ for which we can find a value of τ.

Selecting on *Y*_1_ affects the joint dependence between these variables. To this consider Appendix 2—table 1, the correlation matrix among *X*, *Z*, *Y*_1_, and *Y*_2_ *without* selecting on *Y*_1_. Contrast this with A1, the correlation matrix among *X*, *Z*, *Y*_1_, and *Y*_2_ *with* selecting on *Y*_1_.

(A1) $$\tilde{\Sigma }=\left[\begin{array}{cc} \;{\tilde{\Sigma }}_{12} & Cov\left\{(Z,X,Y_{2}),Y_{1}|Y_{1}>a\right\}\\ Cov\left\{(Z,X,Y_{2}),Y_{1}|Y_{1}>a\right\} & Var(Y_{1}|Y_{1}>a) \end{array}\right]$$

Where:

(14) $${\tilde{\Sigma }}_{12}=\Sigma_{12}+\frac{\phi \left(-\alpha \right)}{\Phi \left(-\alpha \right)}\left(\alpha -\frac{\phi \left(-\alpha \right)}{\Phi \left(-\alpha \right)}\right)\rho \rho^{T}$$

$$\Sigma_{12}=\left[\begin{array}{ccc} 1 & \lambda_{z} & \alpha_{x}+\alpha_{x}\lambda_{z}\\ \lambda_{z} & 1 & \alpha_{x}+\alpha_{x}\lambda_{z}\\ \alpha_{z}+\alpha_{x}\lambda_{x} & \alpha_{x}+\alpha_{z}\lambda_{z} & 1 \end{array}\right]$$

(15) $$Var\left(Y_{1}|Y_{1}>a\right)=\left\{1+\frac{\phi \left(-a\right)}{\Phi \left(-a\right)}\left(a-\frac{\phi \left(-a\right)}{\Phi \left(-a\right)}\right)\right\}\sigma_{y,1}^{2}$$

(16) $$Cov\left\{\left(Z,X,Y_{2}\right),Y_{1}|Y_{1}>a\right\}=\left\{1+\frac{\phi \left(-a\right)}{\Phi \left(-a\right)}\left(a-\frac{\phi \left(-a\right)}{\Phi \left(-a\right)}\right)\right\}\rho$$

Let $a_{0}=\frac{\phi \left(-\alpha \right)}{\Phi \left(-\alpha \right)}\left(\alpha -\frac{\phi \left(-\alpha \right)}{\Phi \left(-\alpha \right)}\right)$, $a_{1}=\alpha_{z}+\alpha_{x}\lambda_{z},a_{2}=\alpha_{x}+\alpha_{z}\lambda_{z}$, $\rho_{1}=\beta_{z}+\beta_{x}\lambda_{z}$, $\rho_{2}=\beta_{x}+\beta_{z}\lambda_{z}$, and $\rho_{3}=(\alpha_{z}+\alpha_{x}\lambda_{z})\beta_{z}(\alpha_{x}+\alpha_{z}\lambda_{z})$

$${\tilde{\Sigma }}_{12}=\left[\begin{array}{ccc} 1+a_{0}\rho_{1}^{2} & \lambda_{z}+a_{0}\rho_{1}\rho_{2} & a_{1}+a_{0}\rho_{1}\rho_{3}\\ \lambda_{z}+a_{0}\rho_{1}\rho_{2} & 1+a_{0}\rho_{2}^{2} & a_{2}+a_{0}\rho_{2}\rho_{3}\\ a_{1}+a_{0}\rho_{1}\rho_{3} & a_{2}+a_{0}\rho_{2}\rho_{3} & 1+a_{0}\rho_{3}^{2} \end{array}\right]$$

From the elements of Appendix 2—table 1 and , we can derive the elements of Appendix 2—table 2, which shows the squared (partial or zero-order) correlation and (partial or univariable) slope of the regression of *Y*_2_ on *X* with and without controlling for *Z* and with and without selecting on *Y*_1_.

**Appendix 2—table 2.** The squared correlation and slope of regression.

Quantity of interest	Without selection on Y_1_	With selection on Y_1_
Squared (zero-order) correlation of X and Y_2_	${\left(\lambda_{z}\alpha_{z}+\alpha_{x}\right)}^{2}$	${\left(\frac{{\tilde{\sigma }}_{23}}{\sqrt{{\tilde{\sigma }}_{22}}\sqrt{{\tilde{\sigma }}_{33}}}\right)}^{2}$
Squared (partial) correlation of X and Y_2_, controlling for Z	$\frac{{\left(-{\tilde{\sigma }}_{23}\right)}^{2}}{{\tilde{\sigma }}_{22}{\tilde{\sigma }}_{33}}$	${\left(\frac{{\tilde{\sigma }}_{23}^{*}}{\sqrt{{\tilde{\sigma }}_{22}^{*}}\sqrt{{\tilde{\sigma }}_{33}^{*}}}\right)}^{2}$
Slope of univariable regression of Y_2_ on X	$\left(\lambda_{z}\alpha_{z}+\alpha_{x}\right)$	$\frac{{\tilde{\sigma }}_{23}}{{\tilde{\sigma }}_{22}}$
Partial slope of regression of Y_2_ on X, controlling for Z	$\frac{\alpha_{x}+\alpha_{x}\lambda_{z}^{2}}{1-\lambda_{z}^{2}}$	$\left(\frac{\sqrt{{\tilde{\sigma }}_{33}}}{\sqrt{{\tilde{\sigma }}_{22}}}\right)\left(\frac{{\tilde{\rho }}_{23}-{\tilde{\rho }}_{13}{\tilde{\rho }}_{12}}{1-{\tilde{\rho }}_{12}^{2}}\right)$

${\tilde{\sigma }}_{12},{\tilde{\sigma }}_{13},{\tilde{\sigma }}_{23},{\tilde{\sigma }}_{11},{\tilde{\sigma }}_{22},{\tilde{\sigma }}_{33}$ are the entries of the matrix ${\tilde{\Sigma }}_{12}$ and $\tilde{\rho }$ are entries of the correlation matrix obtained from ${\tilde{\Sigma }}_{12}$ .

When $\alpha_{x}=0,$ then $a_{1}=\alpha_{z}$, $a_{2}=\alpha_{z}\lambda_{z}$, $\rho_{3}=\alpha_{z}\rho_{1}$, ${\tilde{\sigma }}_{12}=\lambda_{z}+a_{0}\rho_{1}\rho_{2}$

$${\tilde{\sigma }}_{13}=\alpha_{z}+a_{0}\alpha_{z}\rho_{1}^{2},{\tilde{\sigma }}_{23}=\alpha_{z}\left(\lambda_{z}+a_{0}\rho_{1}\rho_{2}\right),{\tilde{\sigma }}_{11}=1+a_{0}\rho_{1}^{2},$$

$${\tilde{\sigma }}_{22}=1+a_{0}\rho_{2}^{2},{\tilde{\sigma }}_{33}=1+a_{0}\alpha_{z}^{2}\rho_{1}^{2}$$

Namely,

$${\tilde{\rho }}_{23}={\left(\frac{{\tilde{\sigma }}_{23}}{\sqrt{{\tilde{\sigma }}_{22}}\sqrt{{\tilde{\sigma }}_{33}}}\right)}^{2},{\tilde{\rho }}_{13}={\left(\frac{{\tilde{\sigma }}_{13}}{\sqrt{{\tilde{\sigma }}_{11}}\sqrt{{\tilde{\sigma }}_{33}}}\right)}^{2},{\tilde{\rho }}_{12}={\left(\frac{{\tilde{\sigma }}_{12}}{\sqrt{{\tilde{\sigma }}_{11}}\sqrt{{\tilde{\sigma }}_{22}}}\right)}^{2}$$

and ${\tilde{\sigma }}_{23}^{*},{\tilde{\sigma }}_{22}^{*},{\tilde{\sigma }}_{33}^{*}$ are coming from the inverse of ${\tilde{\Sigma }}_{12}$

$${\tilde{\sigma }}_{23}^{*}=\lambda_{z}a_{1}+a_{0}\lambda_{z}\rho_{1}\rho_{2}+a_{0}a_{1}\rho_{1}\rho_{3}-a_{2}-a_{0}\rho_{2}\rho_{3}-a_{0}a_{2}\rho_{1}^{2}$$

$$=a_{0}\lambda_{z}\rho_{1}\rho_{2}+a_{0}\alpha_{z}^{2}\rho_{1}^{2}-a_{0}\alpha_{z}\rho_{1}\rho_{2}-a_{0}\alpha_{z}\lambda_{z}\rho_{1}^{2}$$

$${\tilde{\sigma }}_{22}^{*}=1+a_{0}\rho_{1}^{2}+a_{0}\rho_{3}^{2}-a_{1}^{2}-2a_{0}a_{1}\rho_{1}\rho_{3}$$

$$=1+a_{0}\rho_{1}^{2}-\alpha_{z}^{2}-a_{0}\alpha_{z}^{2}\rho_{1}^{2}$$

$${\tilde{\sigma }}_{33}^{*}=1+a_{0}\rho_{1}^{2}+a_{0}\rho_{2}^{2}-\lambda_{z}^{2}-2a_{0}\lambda_{z}\rho_{1}\rho_{2}$$

In all, our derivations show that selecting on *Y*_1_ can have some impact on the causal estimate of the effect of covariate *X* on *Y*_2_. To bring our point home, we perform a simulation study where we randomly select a data set of 1000 according to our data generating above. We consider the eight pairs (*a*, *b*) discussed above, and for each pair, we chose two values of τ (high and low). To each value of τ, we have an associated value of $\lambda_{z},\beta_{x},\beta_{z}$ . We choose the value of $\alpha_{x}=0$ and $\alpha_{z}=0.6$. The sample size for each data set simulated is 50,000 and we report the average bias for the adjusted (controlling for the confounder *Z*) and unadjusted causal effect of the covariate *X* on the response $Y_{2}$ based on the full data (All Data) and the data obtained after selecting on $Y_{1}\left(SelectonY_{1}>\tau \right)$. We report our findings in Appendix 2—table 3. Adjusting for the confounder yields an unbiased estimate of the causal effect of *X* on $Y_{2.}$ Under both full and selected data scenarios, that estimated effect is biased. However, the estimated causal effect is biased for the full data and unbiased for the selected data when omitting the confounder.

**Appendix 2—table 3.** Estimated Average Bias of $\alpha_{x}$ Under Various Scenarios. Where $\tau ,\beta_{x},\beta_{z}$ are selected to Induce a Zero Correlation Between X and Z After Selecting on $Y_{1}$ . Results are based on sample size of n=50,000 and 1000 samples obtained from the data-generating model described above.

a	b	$\lambda_{z}\left(max\right)$	$\beta_{x}$	$\beta_{z}$	$\lambda_{z}$	τ	All data		Select on $Y_{1}>\tau$	
							$\alpha_{x.z}$	$\alpha_{x}$	$\alpha_{x.z}$	$\alpha_{x}$
0.2	0.2	0.0422	0.2000	0.1952	0.0200	–0.5774	–0.0000	0.0118	–0.0002	–0.0002
0.2	0.2	0.0422	0.1999	0.1946	0.0350	1.3809	–0.0001	0.0208	–0.0004	–0.0008
0.2	0.9	0.5519	0.1931	0.8361	0.2700	0.4647	–0.0001	0.1620	–0.0002	–0.0001
0.2	0.9	0.5519	0.1857	0.8175	0.4000	1.8276	–0.0001	0.2398	–0.0002	–0.0003
0.9	0.2	0.2277	0.8936	0.0683	0.1200	0.6717	0.0001	0.0721	0.0005	0.0009
0.9	0.2	0.2277	0.8842	0.0598	0.1900	3.0584	0.0001	0.1140	–0.0080	–0.0118
0.9	0.9	0.5156	0.8710	0.2383	0.2600	–0.1627	–0.0002	0.1556	–0.0001	–0.0003
0.9	0.9	0.5156	0.8207	0.1818	0.4500	2.3590	–0.0002	0.2699	0.0011	–0.0005
